# Psychoactive Medication and Traffic Safety

**DOI:** 10.3390/ijerph6031041

**Published:** 2009-03-10

**Authors:** Joris C. Verster, Monique A.J. Mets

**Affiliations:** Utrecht University, Utrecht Institute for Pharmaceutical Sciences, Faculty of Science, Psychopharmacology Section, P. O. Box 80082, 3508TB Utrecht, The Netherlands; E-Mail: m.a.j.mets@uu.nl

**Keywords:** Driving, drugs, psychoactive medication, traffic safety, ICADTS

## Abstract

Driving a car is important to maintain independence and participate in society. Many of those who use psychoactive medication are outpatients and are thus likely to drive a vehicle. Most common adverse effects that impair driving are reduced alertness, affected psychomotor functioning and impaired vision. This review discusses the effects on driving ability of most commonly prescribed psychoactive drugs, including hypnotics, antidepressants, antihistamines, analgesics and stimulant drugs. Within these categories of medicines significant differences concerning their impact on driving ability are evident. The International Council on Alcohol, Drugs and Traffic Safety (ICADTS) categorization can help physicians to make a choice between treatments when patients want to drive a car.

## 1. Introduction

Psychoactive drugs, i.e. drugs that exert their activity on the Central Nervous System, and drugs that affect motor function are of concern when it comes to traffic safety. Since the vast majority of those who use psychoactive medication are outpatients, it is reasonable to assume that they also participate in traffic. Roadside surveys estimate the incidence of drivers who are under the influence of psychoactive drugs at between 5% and 35% [1]. Given the worldwide increase in prescribing of psychoactive medication [2], traffic safety is an issue that’s becoming increasingly relevant. Yearly, increasing numbers of traffic deaths are reported. Although in the U.S.A. and Europe a significant reduction in traffic accidents is evident, in other parts of the world (e.g. Africa and Southeast Asia) the number of traffic accidents has increased dramatically. In this context, the World Health Organization dedicated the 2004 World Health Day to road safety [3]. This review updates on the effects on driving performance of the most commonly prescribed psychoactive drugs.

## 2. Methodology

**Figure 1.**
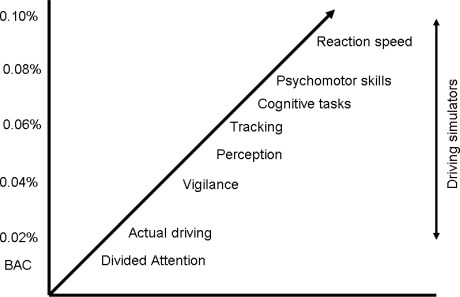
Skills and abilities related to driving and corresponding blood alcohol concentrations at which more than half of behavioral tests show significant impairment [5].

There are various methods to examine driving ability and assess the effects of psychoactive medication on traffic safety. Epidemiological studies provide evidence about the (increased) risk of becoming involved in traffic accidents when using psychoactive medication. Although this is important information, it is gathered after accidents have happened. Ideally, one would like to have this information beforehand in order to prevent driving under the influence of these drugs. A limitation of most epidemiological studies is that the statistical analysis is based on groups of drugs instead of individual drugs. This is unfortunate, because within drug groups the effects of individual drugs on driving ability can differ significantly. Many researchers use laboratory tests to examine driving related skills and abilities such as reaction speed, working memory and psychomotor functioning. Although these skills and abilities are all of great importance to operating a vehicle it has been proven that it is very difficult to predict actual driving performance from these tests [4]. This is caused by the fact that these skills and abilities are tested in isolation, whereas in real driving they are integrated and performed simultaneously. Also, the extent of impairment of individual skills and abilities differs greatly after administration of a psychoactive drug [5]. This is illustrated by Figure 1, showing the blood alcohol concentrations (BAC) at which different skills and abilities become impaired.

Driving simulators are popular to test driving skills. They are safe because no real traffic is involved and tests can be performed in a controlled environment. Traditional driving simulators were often very simple divided attention tasks. Equipment regularly consisted of a steering wheel and a computer screen. Subjects had to perform a tracking task and reaction speed task simultaneously, mimicking two important driving skills. Unfortunately, no other traffic was involved and often no road scenery was depicted on the computer screen. Therefore, these driving simulators had little predictive validity for real driving [6]. The vital lacking element of other traffic has been introduced in most current driving simulators. Equipment of these sophisticated driving simulators often comprises a real car, a wide screen, and road scenery involving other traffic that interacts with the subject. This set-up is a great improvement when compared to the first generation of driving simulators. Nevertheless, it remains to be determined to what extent driving simulators predict actual driving in real traffic. Subjects who perform a driving simulator test are aware of the artificial environment and this may have a significant impact on their driving style and performance.

Given legislative restrictions of most countries, relatively few studies have been performed in real traffic. Methods to determine driving performance were often limited to subjective ratings of driving instructors or researchers and self reports by patients. The subjective nature of these measurements makes it difficult to compare different drugs or dosages. To establish this, objective measurement of the magnitude of impairment is essential. One test that does measure driving performance objectively is the standardized on-the-road driving test in real traffic. Over the past 30 years, many psychoactive drugs have been examined using this test. The methodology of the driving test, applied only in The Netherlands to examine psychoactive medication, will be described below and results from studies applying this test are summarized in this review.

### 2.1. The On-the-Road Driving Test

**Figure 2.**
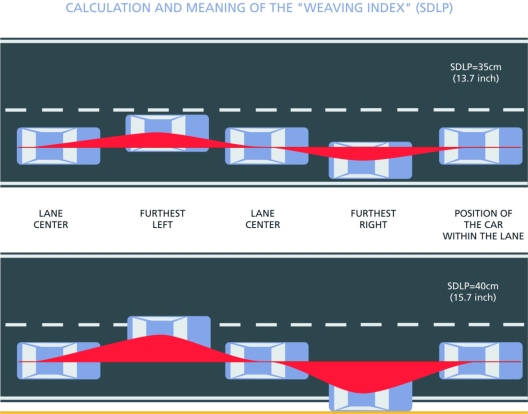
Standard Deviation of the Lateral Position, SDLP. Increased weaving of the car (higher SDLP values) represents reduced vehicle control and may result in out of lane excursions.

The on-the-road driving test in real traffic was developed in the 1980s [7] and has been applied in over 50 studies to determine the effects of psychoactive drugs on driving ability. The test has been highly standardized and has shown to be sensitive to dose-dependent impairment after administration a variety of psychoactive drugs including hypnotics, anxiolytics, antidepressants, analgesics, stimulants, and antihistamines. In the standardized driving test, subjects are instructed to drive a car over a 100-km (61 miles) highway while maintaining a constant speed (58 miles/h) and a steady lateral position within the right (slower) traffic lane. The primary parameter of the test is the Standard Deviation of Lateral Position (SDLP, cm): the weaving of the car. This is shown in Figure 2. It is evident from this Figure that SDLP represents the amount of vehicle control. Higher SDLP values represent increased weaving of the car.

**Figure 3.**
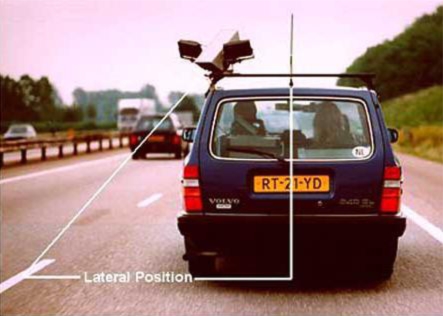
The instrumented car. Note that the camera for lateral position measurements is equipped with two infrared lights, to enable recording during the night and dark weather circumstances. Adapted with permission from reference [12].

A camera, mounted on the roof of the car, continuously records the position of the car within the right traffic lane, by tracking the relative distance of the car from the delineation in the middle of the road. This is illustrated in Figure 3. In the right front seat, a licensed driving instructor accompanies the subject. His main responsibility is to guard safety during the driving test, and he is equipped with a brake and clutch system. If the subject or the driving instructor judges that it is unsafe to continue driving, the test is terminated before completion and the driving instructor transports the subject back to the Institute.

### 2.2. ICADTS Categorization

The categorization system of the International Council on Alcohol, Drugs and Traffic Safety (ICADTS) will be used to indicate whether or not it is safe to drive a car when using a specific psychoactive drug [8]. Drugs are allocated to one of the following categories:

1. Presumed to be safe or unlikely to produce an effect;
2. Likely to produce minor or moderate adverse effects;
3. Likely to produce severe effects or presumed to be potentially dangerous.

To make the categories understandable, a comparison with blood alcohol concentration (BAC) is made. Driving impairment for the categories I, II and III are equivalent to BAC < 0.5 g/L (<0.05%), BAC 0.5–0.8 g/L (0.05−0.08%), and BAC > 0.8 g/L (>0.08%), respectively. Description and interpretation of the categories is summarized in Box 1.

**Box 1. Description of ICADTS category Interpretation and practical use.**

**Category I: Presumed to be safe or unlikely to produce an effect**

In various experimental circumstances negligible or no impairment of driving performance or performance related to driving is repeatedly demonstrated. Also for medicinal drugs that are presumed not to be dangerous based on their pharmacological profile, even though there are no experimental studies that support this presumption. For the most frequently used drugs in this category the effect has been assessed in over-the-road driving tests as equivalent to blood alcohol concentrations < 0.5 g/L (<0.05%).

Advice for the patient: Be careful not to drive before having read the warnings in the package insert.

**Category II: Likely to produce minor or moderate adverse effects**

Some impairment of driving performance or performance related to driving is seen in various experimental laboratory circumstances. Also for drugs that will not produce severely adverse effects, but because of a lack of sufficient experimental studies it can not be established if the effect is moderate, light or absent. For the most frequently used drugs in this category the effect has been assessed in over-the-road driving tests as equivalent to blood alcohol concentrations 0.5–0.8 g/L (0.05–0.08%).

Advice for the patient: Do not drive without consulting a healthcare professional about the possible impairing effects.

**Category III: Likely to produce severe effects or presumed to be potentially dangerous**

In various experimental circumstances gross impairment of driving performance, or performance related to driving, is repeatedly seen. Also for drugs presumed to be potentially dangerous based upon their pharmacological profile, but there are not sufficient experimental studies to support this presumption. For the most frequently used drugs in this category the effect has been assessed in over-the-road driving tests as equivalent to blood alcohol concentrations > 0.8 g/L (>0.08%).

Advice for the patient: Do not drive when this drug is taken and consult a healthcare professional when to start driving again after evaluation of the treatment outcomes.

The effect of different BAC levels on driving performance was determined in 24 social drinkers [9]. A dose-dependent impairment was observed. SDLP increments after alcohol consumption corresponding to the most common legal limits for driving were +2.4 cm (0.05%), +4.1 cm (0.08%), and +5.3 cm (0.10%) and are often used as reference values to illustrate driving safety when using psychoactive drugs. The study revealed a steady correlation between BAC and SDLP.

## 3. CNS Drugs and Traffic Safety

The following sections discuss the effects on driving ability of the most commonly used psychoactive drugs, including hypnotics, anxiolytics, antidepressants, antihistamines, analgesics and stimulant drugs.

### 3.1. Hypnotics/Sleep Medication

**Figure 4.**
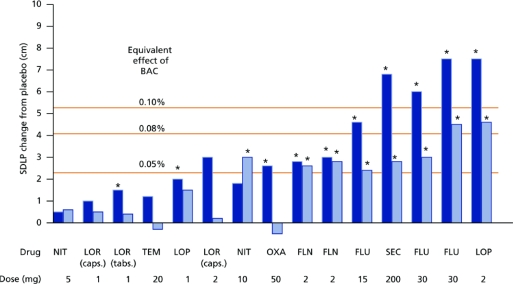
Benzodiazepine hypnotics and driving performance. Standard Deviation of Lateral Position (SDLP) increments relative to placebo are shown. Driving tests were performed in the morning (dark blue bars) and afternoon (light blue bars) (10–11 and 16–17 h after bedtime administration, respectively). Significant differences from placebo are indicated by an asterisk, orange lines indicate levels of SDLP increment observed with most common legal blood alcohol limits for driving a car. NIT, Nitrazepam; LOR, lormetazepam; TEM, temazepam; LOP, loprazolam; FLN, flunitrazepam; FLU, flurazepam, SEC = secobarbital, caps = capsules, tabs = tablets, BAC = blood alcohol concentration.

Several studies have examined the residual effects of benzodiazepine hypnotics on driving ability [10–12]. In these studies, hypnotic drugs were taken at bedtime for 1 or 2 nights. The driving tests were performed the following morning (10–11 hours after intake) and in the afternoon (16–17 hours after intake), corresponding to the times one drives to and from work. Increment relative to placebo for benzodiazepine hypnotics are shown in Figure 4.

Figure 4 shows that benzodiazepine hypnotics significantly impair driving performance. Driving impairment was most pronounced in the morning. In the afternoon, driving impairment was less evident and absent for short-acting benzodiazepines. For long-acting benzodiazepines driving was also impaired in the afternoon; especially when using higher dosages than recommended. To illustrate the magnitude of driving impairment, effects of different dosages of alcohol are also depicted in Figure 3. Most benzodiazepine hypnotics were categorized in ICADTS category II or III (see Table 1).

The Z-drugs zopiclone, zolpidem and zaleplon were developed to overcome the unwanted residual effects of benzodiazepine hypnotics. Unfortunately, the introduction of zopiclone was no improvement. Several on-the-road studies showed pronounced driving impairment after consumption of zopiclone. SDLP increments ranged between 3 and 8 cm, comparable to impairment observed for blood alcohol concentrations of 0.05% to 0.10% (above the legal limit for driving in many countries). Zolpidem, when taken as recommended, has no residual effects on driving ability and thus is a great improvement when compared to benzodiazepines and zopiclone. However, when shortening the time between intake and driving dose-dependent impairment is evident [13]. Also, various accidents and impaired driving have been reported after inappropriate use of zolpidem [14]. Zaleplon has no negative residual effects on driving ability. Even when taken in the middle of the night four hours before driving, twice the recommended dose of zaleplon did not affect driving performance. Results from epidemiological studies confirm that benzodiazepines and zopiclone significantly increase the risk of becoming involved in traffic accidents [10–11,15]. New hypnotics with different mechanisms of action, such as those acting at melatonin or serotonin receptors, are both promising and needed for those patients who have to use hypnotic drugs and want to participate safely in traffic.

### 3.2. Anxiolytics

Up to 50% of the patients visiting their physician suffer from anxiety disorders, including generalized anxiety disorder, panic disorder, post-traumatic stress disorder, obsessive-compulsive disorder, social anxiety disorder, or phobias. A substantial number of those patients use anxiolytics including benzodiazepines, tricyclic antidepressants (TCAs), selective serotonin reuptake inhibitors (SSRIs), or buspirone. Their effects on driving ability have been extensively studied and results supported by epidemiological evidence [16].

Both benzodiazepines and TCAs significantly impaired driving performance after single dose administration. Impairment of benzodiazepines when used as anxiolytic is much more pronounced when compared to impairment when used as hypnotic drug. This difference is caused by the fact that the time between drug intake and the driving test is much greater for hypnotics (10–11 hours) when compared to anxiolytics (1 hour). The different time intervals were chosen to reflect normal use of anxiolytics (during the day, for example after awaking) and hypnotics (at bedtime).

Tolerance develops slowly and after a week of daily treatment with benzodiazepine anxiolytics driving remained significantly impaired [16]. This effect was less pronounced for TCAs. In contrast, SSRIs, 5HT-antagonists and buspirone produced no significant impairment on the driving test after both acute and repeated administration. Corresponding ICADTS categories of most commonly prescribed anxiolytics are summarized in Tables 2 and 3.

Tables 2 and 3 clearly show that benzodiazepine anxiolytics and TCAs (listed as category II and III drugs) are regarded as more dangerous than SSRIs and related compounds (listed as category I drugs).

### 3.3. Antidepressants

The effects of most commonly used antidepressants on driving ability have been investigated applying the on-the-road test [17]. Driving after intake of TCAs (including amitriptyline, doxepine and imipramine), mianserin and mirtazapin was significantly impaired after treatment initiation. Tolerance developed gradually, and after 1 week of treatment driving impairment was absent or much less pronounced. Nocturnal treatment with these antidepressants did not affect next day driving performance. In contrast to the TCAs, SSRIs (including fluoxetine, paroxetine and escitalopram), related antidepressants (venlafaxine and nefazodone), and moclobemide showed no significant effect on driving performance. The ICADTS categorization of most commonly used antidepressant drugs is summarized in Table 3.

### 3.4. Antihistamines

All antihistamines are capable of crossing the blood-brain barrier and thus may cause sedation. Most commonly used antihistamines have been examined using the on-the-road test [18]. Over the past decades 3 generations of antihistamines have been developed, each improving his proceeding generation in terms of less sedation and adverse effects.

The oldest (first-generation) antihistamines (diphenhydramine, triprolidine, terfenadine, dexchlorpheniramine, clemastine) significantly impair driving performance after both one-time and repeated (daily) administration. Second-generation antihistamines (cetirizine, loratadine, ebastine, mizolastine, acrivastine, emedastine, mequitazine) may also impair driving performance, but this differs greatly among individuals. The magnitude and extent of impairment depends on the administered dose, sex, and time between driving and treatment administration. Tolerance develops after four to five days of administration, but impairment is not always absent. In contrast, third-generation antihistamines (fexofenadine, desloratadine, and levocetirizine) produce no driving impairment after both one-time and repeated administration. The ICADTS categorization of most commonly used antihistamines is summarized in Table 4.

### 3.5. Analgesics

Pain itself can significantly impair driving performance [19]. Effective treatment with Non-steroidal anti-inflammatory drugs (NSAIDs) or opioids may (partially) relieve the pain. Up to now, only few driving studies have been performed with analgesics. Laboratory tests of cognitive functioning and psychomotor skills generally do not show significant performance impairment in patients using NSAIDs or acetaminophen. Therefore they are listed in ICADTS category I. One driving study [20] examined the effects on driving of bromfenac. This NSAID, which is no longer marketed, did not affect driving or related skills. The same study also examined the opioid oxycodone. No significant differences from placebo were found, but subjects indicated that much more effort was needed to perform the driving test. Laboratory studies failed to find consistent results when testing opioids [21]. Nevertheless, ICADTS categorizes many opioid analgesics in class II (e.g., oxycodone, and codeine) or III (e.g. morphine, tramadol and fentanyl). Opioids show a strong dose-dependent impairing effect on performance and after treatment initiation dosages are often gradually increased. This may interfere with developing tolerance to their impairing effects, and thus these drugs are often grouped in category II or III.

Chronic pain patients are often treated with antidepressants such as amitriptyline instead of opioids and NSAIDs. Thirteen hours after treatment administration, amitriptyline (25 mg) significantly impaired on-the-road driving performance in chronic neuropathic pain patients [22]. After two weeks of daily use, tolerance developed to the impairing effects of amitriptyline.

### 3.6. Stimulant Drugs

Stimulant drugs are used in the treatment of attention deficit hyperactivity disorder (ADHD) and narcolepsy. Purpose of using these drugs is to improve attention and daytime alertness. Two studies showed improvement of driving performance after stimulant drug use.

Ramaekers and colleagues [23] examined the effects of 3-4-methylendioxymethamphetamine (MDMA) (75 mg), methylphenidate (20 mg) and placebo on driving performance in 18 recreational MDMA users. The on-the-road driving test and a car following test were performed three to five hours after drug use, and the next day (27 to 29 hours after intake) to examine possible withdrawal effects. Both MDMA and methylphenidate significantly improved driving performance as indicated by reduced weaving. However, MDMA negatively affected performance in the car following test, whereas performance after using methylphenidate did not differ significantly from placebo. During withdrawal, no significant differences from placebo were found. Verster and colleagues [24] examined the effects of methylphenidate on driving performance in adults with ADHD. After a training session and withdrawal of methylphenidate for at least four days, patients participated in a double blind trial and performed an on-the-road driving test after intake of placebo or their regular dose of methylphenidate. In line with Ramaekers’ findings, driving performance after using methylphenidate was significantly improved when compared to placebo.

## 4. Conclusions

Various psychoactive drugs affect driving performance. These effects are most prominent after treatment initiation and tolerance develops after chronic use. Impairment further depends on dose and half-life of a drug, time after administration, gender and age.

Limitations of current driving research include the fact that they have not examined driving in patients who chronically use psychoactive medication. Epidemiological data show that after long-term use of psychoactive medication tolerance develops to the impairing effects of these drugs. Patients get used to the adverse effects of drugs, and gradually they wear off as do the risks of traffic accidents [25]. Tolerance develops slowly and is much less likely to develop after intermittent (as-needed) use. For example, increased traffic accident risks for users of benzodiazepine hypnotics have been reported after one year of chronic use [10,11]. Unfortunately, on-the-road studies have focused primarily on short term use (i.e. one day to two weeks). One study did examine the effects of four weeks daily treatment with diazepam [26] and confirmed that tolerance develops slowly. After four weeks of treatment with diazepam SDLP increment was still significantly increased. Nevertheless, epidemiological studies have shown no significant increase in traffic accident risk after chronic use (> 1 year) of other psychoactive drugs such as opioids [27].

A second limitation is that individual differences between patients are often not taken into account. Most drugs are supplied in a standardized dose, not taking into account age, gender and metabolism of individual users. However, these factors are important in determining the presence and magnitude of adverse effects. In some driving studies – but not in general, it has been shown that SDLP increment in women is significantly greater than in men [28]. Also, elderly often perform worse when compared to healthy young adults [29]. In this context, it is unfortunate that most experimental studies have been conducted in healthy male young adults, whereas patients using psychoactive medication are often female elderly.

Future pharmaceutical research should focus on developing new psychoactive medication that produces less sedation and adverse effects. These new drugs should be tested preferably in healthy volunteers followed by studies in patients who actually need the medication. Effects on driving ability after long-term use should be examined as well.

Finally, for many diseases a number of different treatment options are available. In terms of traffic safety, physicians should choose medication that has shown to be devoid of impairing effects on driving ability. The ICADTS categorization can help them in making this decision.

## Disclaimer

Although the information presented below has been gathered and evaluated with great care, the authors will not accept any liability after use of the information by patients taking the medicines discussed. Patients should always consult their physician concerning whether or not it is safe to drive a car.

## Figures and Tables

**Table 1.** ICADTS classification of commonly prescribed hypnotics and sedative drugs [8].

Substance name	Category
***Barbiturates***	
Secobarbital	III

***Benzodiazepine derivatives***	

Flurazepam	III
Nitrazepam	III
Flunitrazepam	III
Estazolam	III
Triazolam	III
Lormetazepam	III
Temazepam	III
Midazolam	III
Brotizolam	III
Quazepam	III
Loprazolam	III
***Benzodiazepine related drugs***	
Zopiclon	III
Zolpidem	II

**Table 2.** ICADTS classification of anxiolytic drugs [8].

Substance name	Category
***Benzodiazepine derivatives***	
Diazepam	III
Chlordiazepoxide	III
Medazepam	II
Oxazepam	III
Lorazepam	III
Bromazepam	III
Clobazam	II
Ketazolam	III
Alprazolam	III
***Azaspirodecandione derivatives***	
Buspirone	I

**Table 3.** ICADTS classification of commonly prescribed antidepressants [8].

Substance name	Category
***Non-selective monoamine reuptake inhibitors***	
Desipramine	II
Imipramine	II
Clomipramine	II
Amitriptyline	III
Nortriptyline	II
Doxepin	III
***Selective serotonin reuptake inhibitors***	
Fluoxetine	I
Citalopram	II
Paroxetine	I
Sertraline	II
Fluvoxamine	II
Escitalopram	II
***Monoamine oxidase A inhibitors***	
Moclobemide	II
***Other antidepressants***	
Mianserin	III
Trazodone	III
Nefazodone	II
Mirtazapine	III
Venlafaxine	I
Reboxetine	I

**Table 4.** ICADTS classification of commonly prescribed antihistamines [8].

Substance name	Category
***Aminoalkyl ethers***	
Diphenhydramine	III
Clemastine	III
***Substituted alkylamines***	
Dexchlorpheniramine	II
Chlorphenamine	II
Pheniramine	II
***Phenothiazine derivatives***	
Promethazine	III
Mequitazine	II
***Piperazine derivatives***	
Meclozine	II
Cetirizine	II
Levocetirizine	I
***Other antihistamines for systemic use***	
Triprolidine	III
Terfenadine	I
Loratadine	I
Azelastine	I
Ebastine	I
Mizolastine	II
Fexofenadine	I
Desloratadine	I
